# Grain-sized moxibustion activates dendritic cells to enhance the antitumor immunity of cancer vaccines

**DOI:** 10.1186/s13020-025-01134-w

**Published:** 2025-05-27

**Authors:** Weiming Shen, Dan Hu, Chenyuan Gong, Cheng Fang, Jiaojiao Luo, Lixin Wang, Chao Yao, Huangan Wu, Chen Zhao, Shiguo Zhu

**Affiliations:** 1 Department of Immunology and Pathogenic Biology, School of Integrative Medicine, Shanghai University of Traditional Chinese Medicine, 1200 Cai Lun Road, Shanghai, 201203 People’s Republic of China; 2 Center for Traditional Chinese Medicine and Immunology Research, Shanghai University of Traditional Chinese Medicine, 1200 Cai Lun Road, Shanghai, 201203 People’s Republic of China; 3 School of Acupuncture, Moxibustion and Tuina, Shanghai University of Traditional Chinese Medicine, 1200 Cai Lun Road, Shanghai, 201203 People’s Republic of China; 4 Yueyang Hospital of Integrated Chinese and Western Medicine, Shanghai University of Traditional Chinese Medicine, Shanghai, 200437 China; 5 State Key Laboratory of Systems Medicine for Cancer, Renji Hospital, Shanghai Jiao Tong University School, Shanghai, 200030 China

**Keywords:** Cancer vaccine, Grain-sized moxibustion, β-adrenergic receptor, CD4^+^ T cells, Dendritic cells

## Abstract

**Background:**

Moxibustion, a traditional Chinese medicine (TCM) therapy, employs thermal stimulation from the combustion of *Artemisia argyi* H.Lév. & Vaniot at acupoints to treat "deficiency-cold syndromes" (*xuhan zheng*), historically linked to immune dysfunction and chronic inflammation. Modern pharmacological studies showed that grain-sized moxibustion (gM) enhances innate immune surveillance such as natural killer (NK) cell recruitment. However, its synergy with vaccine-induced adaptive immunity remains unexplored. Guided by the TCM principle of *fu zheng qu xie* (“fortify the host to dispel pathogens”), this study investigated whether gM augments cancer vaccine efficacy and validate the mechanistic basis of thermal acupoint stimulation in amplifying adaptive antitumor immunity.

**Methods:**

In tumor-bearing mice model, gM was applied to the ST36 (Zusanli) acupoint. Adjuvant effects on the cancer vaccine were evaluated through flow cytometry, β-adrenergic receptor blockade, and cell depletion.

**Results:**

gM synergized with the cancer vaccine, significantly suppressing tumor growth. Mechanistically, gM inhibited β-adrenergic signaling, driving DC maturation and subsequent coordination of CD4^+^ T cell, CD8^+^ T cell and NK cell responses. CD4^+^ T cells as primary effectors, with NK cells playing a secondary role. Propranolol mirrored gM’s effects, further enhancing DC activation and tumor suppression when combined with vaccination.

**Conclusion:**

Both gM and β-blockers enhance cancer vaccine efficacy through β-adrenergic suppression and maturation of DC. These findings mechanistically bridge TCM’s *fu zheng qu xie* strategy with modern immunotherapy, positioning β-adrenergic modulation as a convergent target for traditional and pharmacological interventions.

**Supplementary Information:**

The online version contains supplementary material available at 10.1186/s13020-025-01134-w.

## Introduction

Cancer vaccines aim to prime antigen-specific immunity against tumors by delivering tumor antigens (e.g., inactivated cells, lysates, or peptides) to antigen-presenting cells (APC) [1–6]. Despite preclinical success, clinical efficacy remains limited by inadequate T-cell activation and immunosuppressive tumor microenvironments [7, 8]. Current strategies—adjuvant optimization [9], neoantigen discovery [10, 11], and checkpoint inhibitor combinations [12, 13]—have yet to fully overcome these barriers [14], necessitating innovative approaches that integrate traditional medical wisdom.

Traditional Chinese medicine (TCM) offers a millennia-old framework for immune modulation. Among its modalities, moxibustion—thermal stimulation of acupoints via combustion of *Artemisia argyi* H.Lév. & Vaniot (verified via http://www.theplantlist.org, accessed on 2025-03-04)—has been systematically documented since the *Huangdi Neijing* (475–221 BCE) for treating *xuhan zheng* (“deficiency-cold syndrome”) [15, 16], a condition marked by chronic inflammation and immune dysfunction [17, 18]. TCM theory posits that moxibustion restores balance through dual actions: tonifying host resistance (*zhengqi*) by warming meridians and dispelling pathogenic factors (*xieqi*) via purgation [19]. Modern studies attribute its effects to thermal, radiative, and pharmacological properties of moxa combustion [20], with demonstrated roles in analgesia, immunomodulation, and anti-aging [21–25]. Critically, recent trials highlight moxibustion’s potential in cancer care, particularly for managing chemotherapy-induced fatigue and immune suppression [26, 27].

Grain-sized moxibustion (gM), a refined technique using cone-shaped moxa, was historically prescribed in *Shenji Zonglu* (eleventh century CE) for consumptive diseases (*xulao*) characterized by progressive immune decline—a profile analogous to cancer-related immunosuppression. Building on this legacy, we hypothesize that gM enhances cancer vaccine efficacy through two convergent mechanisms: (1) APC Activation: gM-induced local inflammation may enhance APC maturation and antigen presentation, amplifying T-cell priming; (2) TME Remodeling: By recruiting NK cells and reducing immunosuppressive cytokines (e.g., IL-10, TGF-β), gM could facilitate T-cell infiltration into tumors.

This study explores the theoretical interface between TCM's Fuzheng Quxie principle (reinforcing vital qi and eliminating pathogenic factors) and tumor immunology by drawing parallels between Yang-deficiency cold coagulation pathogenesis and the cold tumor phenotype [28, 29]—the former describing TCM's pathological cold accumulation in cancer evolution [30, 31], the latter referring to modern immunology's characterization of immune-excluded malignancies, thereby highlighting the therapeutic relevance of Yang-strengthening therapy in modulating tumor microenvironmental frigidity. Notably, cancer vaccine represents a modern approach to converting immunologically inert ("cold") tumors into immunoreactive ("hot") lesions [29], while gM is as one Yang-strengthening therapy to combat pathological cold accumulation [26, 27, 32]. By integrating gM into cancer vaccine regimens, we establish a bidirectional translation model where ancient therapeutic wisdom informs contemporary immunotherapy.

## Materials and methods

### Cell lines and cell culture

The Lewis Lung Carcinoma (LLC) tumor cells [33] were bought from the Cell Bank of the Chinese Academy of Science and cultured in high-glucose DMEM (SH30243.01, HyClone) supplemented with 10% fetal bovine serum (S1580-500, Biowest) and 1% penicillin‒streptomycin (60162ES76, YEASEN) in a humidified incubator at 37 °C with 5% CO_2_.

### Bone marrow dendritic cell generation

DC were generated from bone marrow progenitors according to a published method [34]. Briefly, bone marrow cells were isolated from the femurs and tibias of 5–6 week-old C57BL/6 mice by flushing with sterile saline solution. The suspension was then filtered through a 70 μm mesh and centrifuged at 1000 rpm for 5 min to collect the cells. The red blood cells was removed with red blood lysis buffer (Biolegend, catalog #420301) for 5 min at room temperature. The remaining cells were suspended and cultured in 100 mm Petri dishes at 5 × 10^5^/mL with 10 mL specialized medium (RPMI1640 supplemented with 10% FBS, 20 ng/mL murine GM-CSF (576304, Biolegend), 10 ng/mL murine IL-4 (574304, Biolegend) and 1% penicillin‒streptomycin). On day 3, the same volume of fresh medium was added to the Petri dish. On day 6, the medium was changed with fresh medium. On day 7, immature DC were harvested and used for the next assays.

### Cancer vaccine preparation

Tumor cell lysate vaccines (TCL) and DC vaccines (DCV) were prepared according to previously established protocols [35]. For the TCL, LLC cells were harvested at a concentration of 2 × 10^7^ cells/mL and transferred to a cryovial. The cells were lysed through three cycles of freeze-thawing, alternating between liquid nitrogen and a 37 °C water bath. Subsequently, the lysate supernatant was obtained by centrifugation at 1000 rpm for 5 min and stored at − 80 °C until further use.

For the DCV, bone marrow-derived dendritic cells were cultured at a density of 1 × 10^6^ cells/mL and co-incubated with 100 μL of TCL (100 μg/mL) for 24 h. After co-culture, the DC were pelleted by centrifugation and then resuspended in 100 μL of phosphate-buffered saline (PBS) within a centrifuge tube, thus forming the DCV preparation.

The cancer vaccine was subcutaneously injected into the upper back of mice (contralateral to the tumour) on days 1, 8 and 15 after tumor modelling [11, 36]. The usage of TCL is 10 μg per mouse and DCV is 1 × 10^6^ dose per mouse.

### Grain-sized moxibustion

Grain-sized moxibustion was prepared and administered following a previously established protocol [26]. The moxa wool (47289, DAYUJP) was meticulously crafted into small cones (diameter 0.8 cm * height 0.9 cm), which were then positioned on the bilateral Zusanli (ST 36) acupoints located on the mice. The ST 36 acupoints are situated 2 mm below and lateral to the anterior tubercle of the tibia. Once in place, the moxa cones were ignited and permitted to burn slowly. The cones were promptly removed upon complete combustion. For the sham group, grain-siezed moxibustion was performed at the mouse tail to avoid other potential effect.

The moxibustion intervention commenced on the day of tumor modeling and was subsequently repeated every other day until the conclusion of the experiment. Each acupoint was treated three times to ensure consistent and effective stimulation and the total treatment time of one acupoint is about 30 s.

### Mice

Male C57BL/6 mice (6 week of age) were purchased from Vital River and bred under specific pathogen-free (SPF) environmental conditions. All procedures were approved by the Institutional Animal Care and Use Committee at Shanghai University of Traditional Chinese Medicine and the methods were performed in accordance with Animal Research: Reporting of In Vivo Experiments (ARRIVE) guidelines and the Guide for the Care and Use of Laboratory Animals published by the National Institutes of Health.

### Subcutaneous transplanted tumor model

A total of 5 × 10^5^ LLC tumor cells were subcutaneously injected into the upper back of the mice. Tumour sizes were monitored every 2 days by using an electronic calliper, and tumour volumes were calculated by the following formula: V = 0.5 × a × b^2^, where V = tumour volume, a = maximum tumour diameter, and b = minimum tumour diameter. After 3 weeks or when the tumour volume reached 2000 mm^3^, the mice were sacrificed by inhalation of CO_2_. The tumours were excised, weighed, and photographed.

### Immune cells depletion

For NK cell depletion, an anti-mouse NK1.1 antibody (PK136, 108702) was intraperitoneally injected (100 μg per mouse) every 7 days [26]. For CD4^+^ T cells and CD8^+^ T cells depletion, anti-CD4 (GK1.5, 100402) and anti-CD8 (2.43, 100,702) antibodies were intraperitoneally injected (100 μg per mouse) every 3 days [37, 38]. Purified Mouse IgG2a, κ Isotype Ctrl Antibody (400202) was used as a control.

### β-adrenoceptor blockade

For β-adrenoceptor blockade, propranolol hydrochloride (Prop) was dissolved in PBS and intraperitoneally injected at a concentration of 15 mg/kg/d beginning on the day of tumor modeling [39]. Propranolol hydrochloride (H32020133) was purchased from Jiangsu Yabang Aipusen Pharmaceutical Co., Ltd. β-AR agonist Epinephrine hydrochloride (E4642) was provided by Sigma. PKA Inhibitor Fragment (6-22) amide TFA (HY-P1290A) and BAY11 (HY-10257) were purchased from MCE.

### Immunophenotype analysis

Tumors, spleens, and tumor-adjacent lymph nodes were isolated from the tumour-bearing mice to measure the levels of T cells, NK cells and DC by flow cytometry [26, 35]. Compared with spleens and lymph nodes, tumours were cut into small pieces with scissors and digested with 0.1% collagenase (40507ES60, Yeasen) for two hours. Then, all tissues were filtered through a 70 μm mesh to make single-cell suspensions. Erythrocytes in tumors and spleens were removed with red blood lysis buffer (420,301, Biolegend). The cells were exposed to appropriate fluorescence-conjugated antibodies at 4 °C for 30 min in the dark and then washed and suspended in PBS containing 1% FBS. The data were obtained with a CytoFLEXLX instrument (Beckman) and analysed with FlowJo software.

### Flow cytometry

For analysis of immune cells in spleen, lymph node and tumour, CD45^+^ cells were firstly sorted from all living cells, and then CD3^+^ T cells and CD3^−^NKp46^+^ NK cells were sorted from CD45^+^ cells. CD4^+^ T helper cells and CD8^+^ cytotoxicity T cells were sorted from CD3^+^ cells. For analysis of DC, CD11c^+^ Cells were sorted from CD45^+^ cells, then MHCII and CD86 expression were analysed in CD11c^+^ cells. To determine the proportion of IFN-γ^+^ CD4^+^ T cells, splenocytes were isolated from mice, and red blood cells were lysed. The remaining cells were counted and co-cultured with LLC cells at a ratio of 3:1 for 6 h. Intracellular cytokine production was blocked using Brefeldin A (BFA, HY-16592, MCE). Following the incubation period, the splenocytes were harvested and stained for CD3 and CD4, as well as intracellular IFN-γ. The frequency of IFN-γ^+^CD4^+^ T cells was then analyzed. To validate that DC can be affected by propranolol, the expression of β-AR also was be detected. The following fluorophore-conjugated antibodies were used: PE/Cyanine7-conjugated anti-mouse CD45 (147704), FITC-conjugated anti-mouse CD4 (100406), PE-conjugated anti-mouse CD3 (100206), PerCP/Cy5.5-conjugated anti-mouse CD8α (100734), APC-conjugated anti-mouse CD335/NKp46 (137608), APC-conjugated anti-mouse CD11c (117309), FITC-conjugated anti-mouse I-A/I-E (MHCII, 107606), PE-conjugated anti-mouse CD86 (105106), APC-conjugated anti-mouse IFN-γ (505810), PE Donkey anti-rabbit IgG Antibody (406421) were purchased from Biolegend Ln. β1 Adrenergic Receptor Polyclonal Antibody (PA5-95742) was purchased from Invitrogen.

### Statistical analysis

All the data are presented as the means ± standard errors of the means (SEM) and were analysed with SPSS statistical software. One-way analysis of variance, two-way analysis of variance and independent-samples t tests were used to assess statistical significance. P value < 0.05 was considered to indicate statistical significance.

## Results

### Grain-sized moxibustion promotes the immune response of cancer vaccines to inhibit tumor growth

**Fig. 1:**
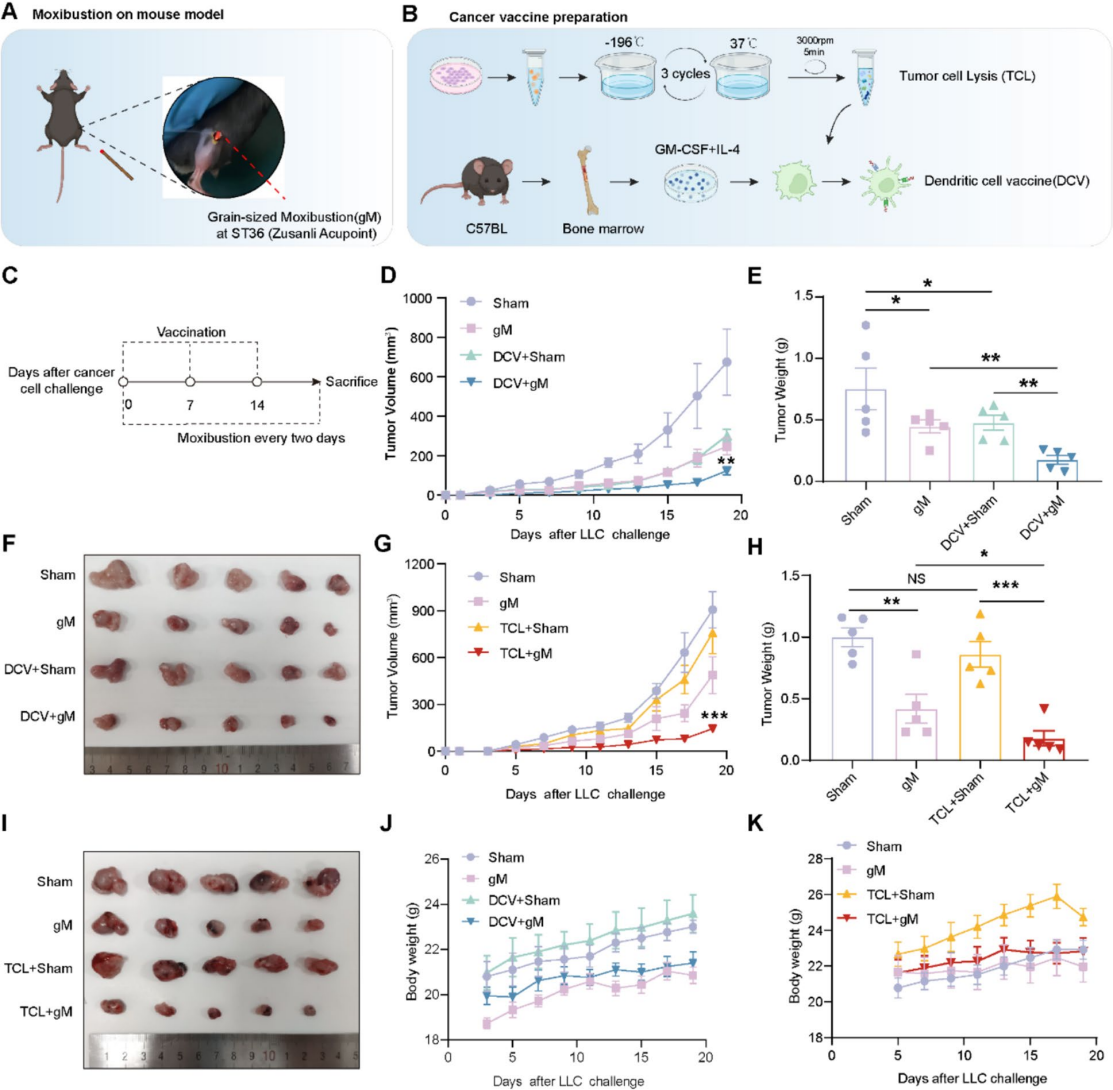
The combination of gM with cancer vaccines improves tumor regression. **A** Grain-sized moxibustion (gM) at ST36 (accupoint zusanli) in a mouse model; **B** Cancer vaccine preparation involving tumor cell lysate (TCL) and dendritic cell vaccine (DCV); **C** Schematic outline of the intervention protocol used in this study. Briefly, 5 × 10^5^ tumor cells were subcutaneously injected on day 0, and mice were subcutaneously immunized on days 0, 7, 14 and treated with gM at ST36 every two days; **D–F** Tumor growth curves/tumor weight/tumor size of mice from the Sham, gM, DCV + Sham and DCV + gM groups (n = 5 mice per group); **G–I** Tumor growth curves/tumor weight/tumor size of mice from the Sham, gM, TCL + Sham and TCL + gM groups (n = 5 mice per group). **J–K** The body weight of mice in different groups. * *p* < 0.05; ** *p* < 0.01; *** *p* < 0.001

In our previous study, we demonstrated that grain-sized moxibustion (gM) stimulation at the ST36 acupoint in tumor-bearing mice could elevate the level of natural killer (NK) cells, thereby inhibiting tumor growth [26]. To sustain the therapeutic effects of gM, we continued to administer stimulation at the ST36 site (Fig. 1A). For the preparation of cancer vaccines, we developed both vaccines: tumor cell lysate vaccines (TCL) and dendritic cell vaccines (DCV) (Fig. 1B). To evaluate the impact of gM on the efficacy of cancer vaccines, we administered the TCL/DCV to mice with lung carcinoma (LLC) at Days 0, 7, and 14, and performed gM every two days starting from Day 0 (Fig. 1C). Our findings indicated that the TCL alone did not significantly inhibit tumor growth compared to the control group, whereas the DCV demonstrated improved efficacy (Fig. 1D and H). When combined with gM, the TCL achieved efficacy comparable to that of the DCV (Fig. 1D–F, H–J), suggesting that gM can enhance the therapeutic efficacy of cancer vaccines, particularly the TCL. With respect to safety, there were no substantial changes in the body weights of mice across all groups (Fig. 1G, K).

**Fig. 2:**
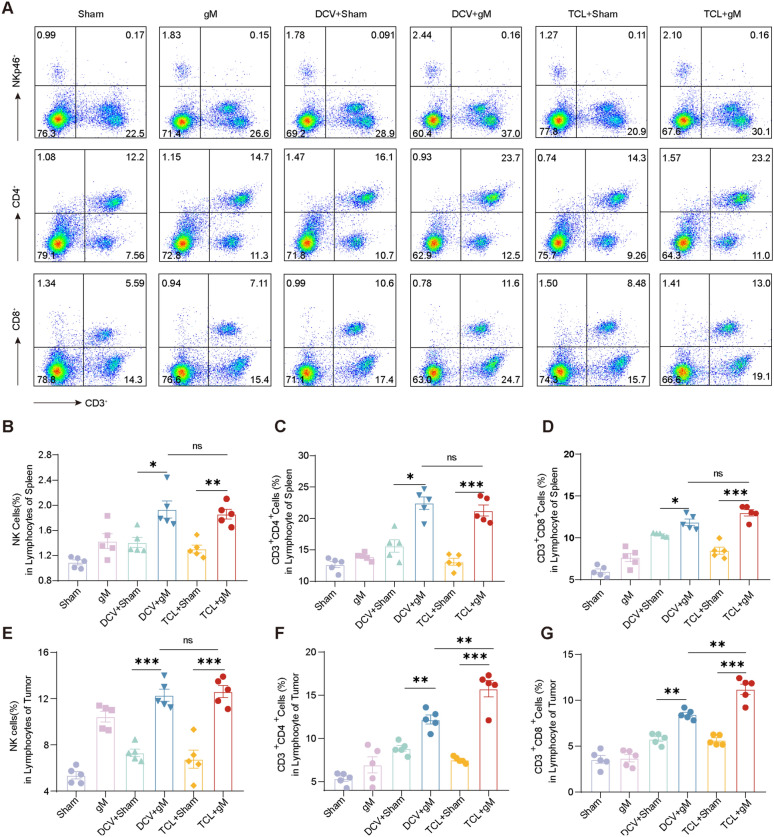
The combination of gM with cancer vaccines enhances the immune response. **A–D** Flow cytometry analysis of NK, CD4^+^T and CD8^+^ T cells in splenic lymphocytes from the Sham, gM, DCV + Sham, DCV + gM, TCL + Sham and TCL + gM groups (n = 5 mice per group); **E–G** Flow cytometry analysis of NK, CD4^+^T and CD8^+^ T cells in tumor lymphocytes from the Sham, gM, DCV + Sham, DCV + gM, TCL + Sham and TCL + gM groups (n = 5 mice per group). “ns”: no statistical significance. * *p* < 0.05; ** *p* < 0.01; *** *p* < 0.001

To investigate whether gM potentiates the anti-tumor effects of cancer vaccines through enhanced immune responses, we assessed the lymphocyte populations in tumor-bearing mice. Our results revealed that the combination of gM with the TCL/DCV significantly increased the levels of NK cells, as well as CD4^+^ and CD8^+^ T cells, both in the spleen (Fig. 2A–D) and the tumor tissue (Fig. 2E–G). To further prove that gM enhances the anti-tumor immune response of cancer vaccines, we also detected IFN-γ secreted by T cells, which is increased in the treatment groups (Figure S1). These findings suggest that gM enhances the anti-tumor immune response of cancer vaccines, with NK cells, CD4^+^, and CD8^+^ T cells potentially contributing to the synergistic tumor regression.

### Grain-sized moxibustion enhances cancer vaccine partly depends on NK cells

**Fig. 3:**
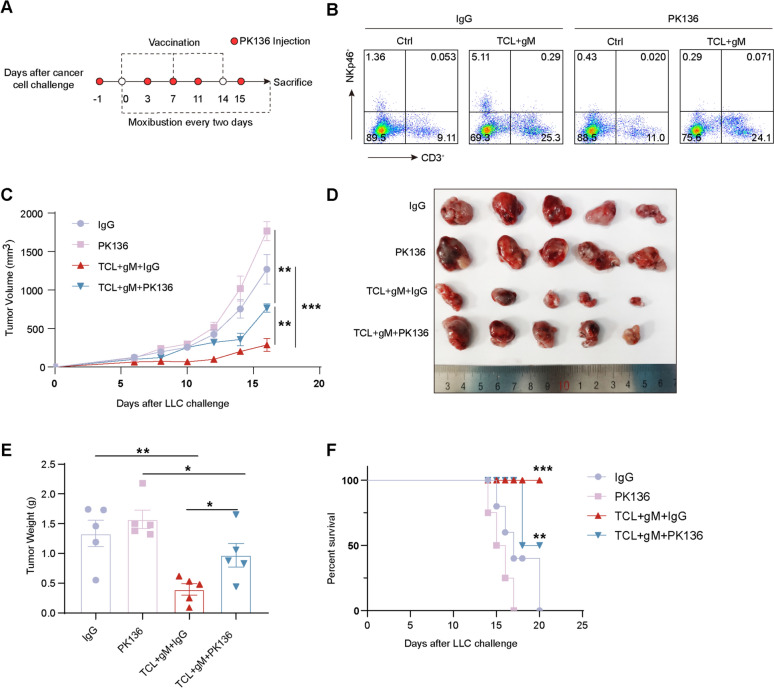
The depletion of NK cells partly reversed the inhibitory effect of gM combined with the TCL vaccine on tumors. **A** Schematic outline of the NK depletion protocol (PK136: anti-NK antibody) used in this study; **B** Flow cytometry analysis of NK cells in splenic lymphocytes from the Ctrl and TCL + gM groups with/without PK136 treatment (n = 5 mice per group); **C–E** Tumor growth curves/tumor size/tumor weight of the IgG, PK136, TCL + gM + IgG and TCL + gM + PK136 groups (n = 5 mice per group). **F**. Survival curves of the IgG, PK136, TCL + gM + IgG and TCL + gM + PK136 groups (n = 5 mice per group). * *p* < 0.05; ** *p* < 0.01; *** *p* < 0.001

Our previous research established that NK cells are the primary effector cells mediating the inhibition of tumor growth by gM treatment [26]. To explore whether gM potentiates the efficacy of cancer vaccines through the action of NK cells, we depleted NK cells in tumor-bearing mice using an anti-PK136 antibody via intraperitoneal injection (Fig. 3A, B). Notably, the depletion of NK cells partially reversed the anti-tumor effects of the combination therapy; however, there remained a statistically significant difference in tumor growth suppression when compared to the group treated with PK136 alone (Fig. 3C–F). These findings suggest that while NK cells contribute to the therapeutic effects of the combination therapy, they do not play an exclusive role. The persistence of anti-tumor activity despite NK cell depletion indicates that other immune cells are likely involved in the mechanism of action of the gM-enhanced cancer vaccine.

### CD4^+^ T cells are the main effector cells in combination therapy

**Fig. 4:**
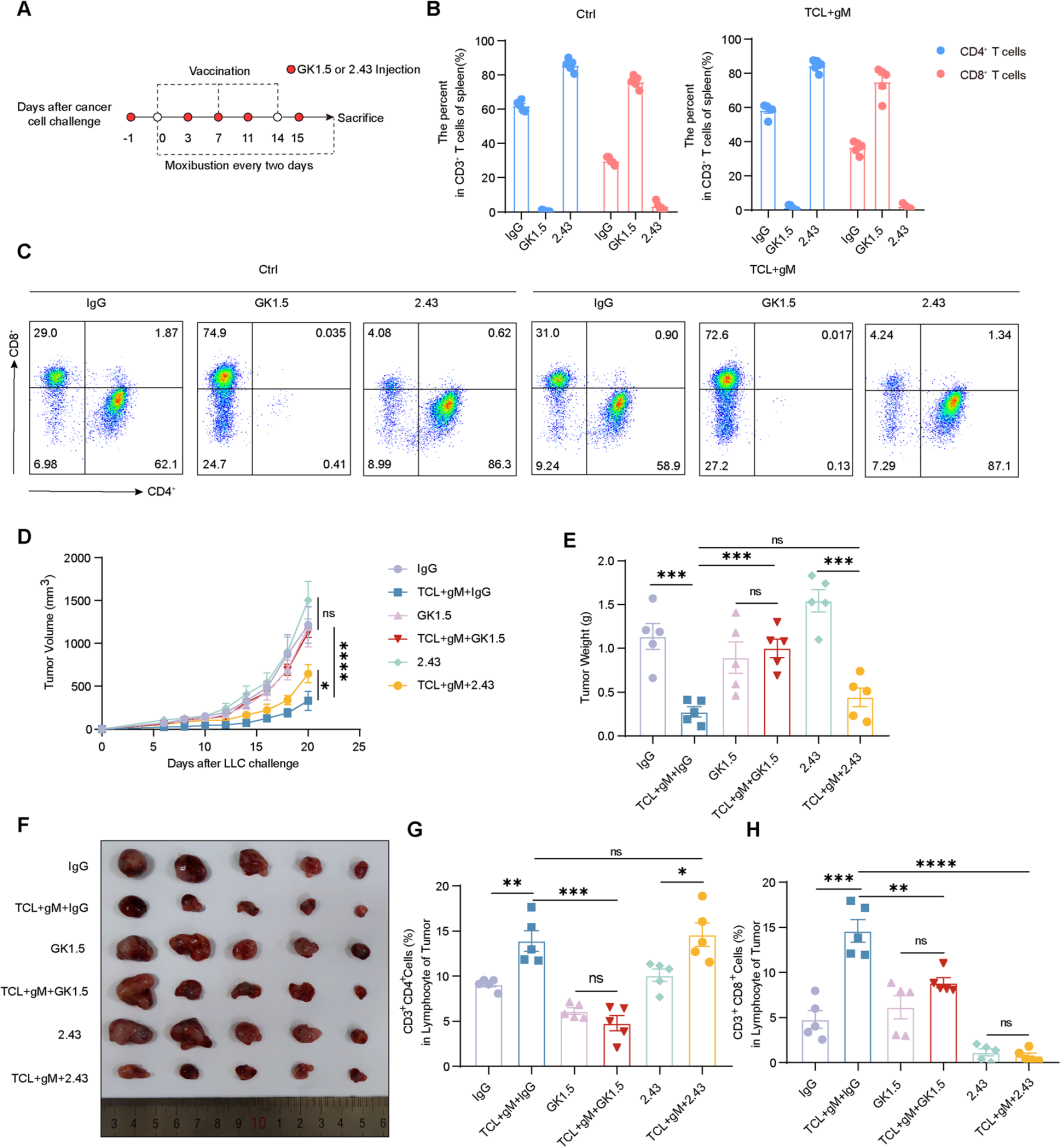
The combination of gM with the TCL vaccine inhibits tumor growth via CD4^+^ T cells but not CD8^+^ T cells. **A** Schematic outline of the CD4^+^ T-cell or CD8^+^ T-cell depletion protocol (GK1.5: anti-CD4 antibody; 2.43: anti-CD8 antibody) used in this study; **B, C** Flow cytometry analysis of CD4^+^/CD8^+^ T cells in splenic lymphocytes from the Ctrl and TCL + gM groups with/without GK1.5/2.43 (n = 5 mice per group); **D–F** Tumor growth curves/tumor Weights/tumor sizes from the Ctrl and TCL + gM groups with/without GK1.5/2.43 (n = 5 mice per group). **G**, **H** Flow cytometry analysis of CD4^+^/CD8^+^ T cells in lymphocytes from tumors from the Ctrl and TCL + gM groups with/without GK1.5/2.43 treatment. “ns”: no statistical significance. * *p* < 0.05; ** *p* < 0.01; *** *p* < 0.001;**** *p* < 0.0001

Numerous studies have highlighted the pivotal role of T cells, particularly CD8^+^ T cells, in the efficacy of cancer vaccines [40]. To delineate the primary effector cells involved, we depleted CD4^+^ and CD8^+^ T cells using the specific antibodies GK1.5 and 2.43, respectively (Fig. 4A, B). In comparison to the IgG control group, the depletion of CD8^+^ T cells led to an increase in tumor growth, highlighting the beneficial role of CD8^+^ T cells in tumor suppression (Fig. 4C–E). However, in the group receiving the combination therapy, the absence of CD8^+^ T cells only partially reversed the anti-tumor effects (Fig. 4C–E). In contrast, the complete reversal of the combination therapy's anti-tumor effect was observed only when CD4^+^ T cells were depleted (Fig. 4C–E). These findings suggest that CD4^+^ T cells may play a more critical role in the therapeutic efficacy of the combination therapy than either NK cells or CD8^+^ T cells.

Furthermore, we analyzed the infiltration of CD4^+^ and CD8^+^ T cells within the tumor microenvironment and found that their levels were consistent with those detected in the spleen (Fig. 4F–G). This suggests a correlation between the peripheral immune response and the cellular dynamics within the tumor, supporting the notion that CD4^+^ T cells are integral to the mechanism of action of the gM-enhanced cancer vaccine.

### Grain-sized moxibustion promotes DC activation in tumor-draining lymph nodes by inhibiting β-adrenergic receptors

**Fig. 5:**
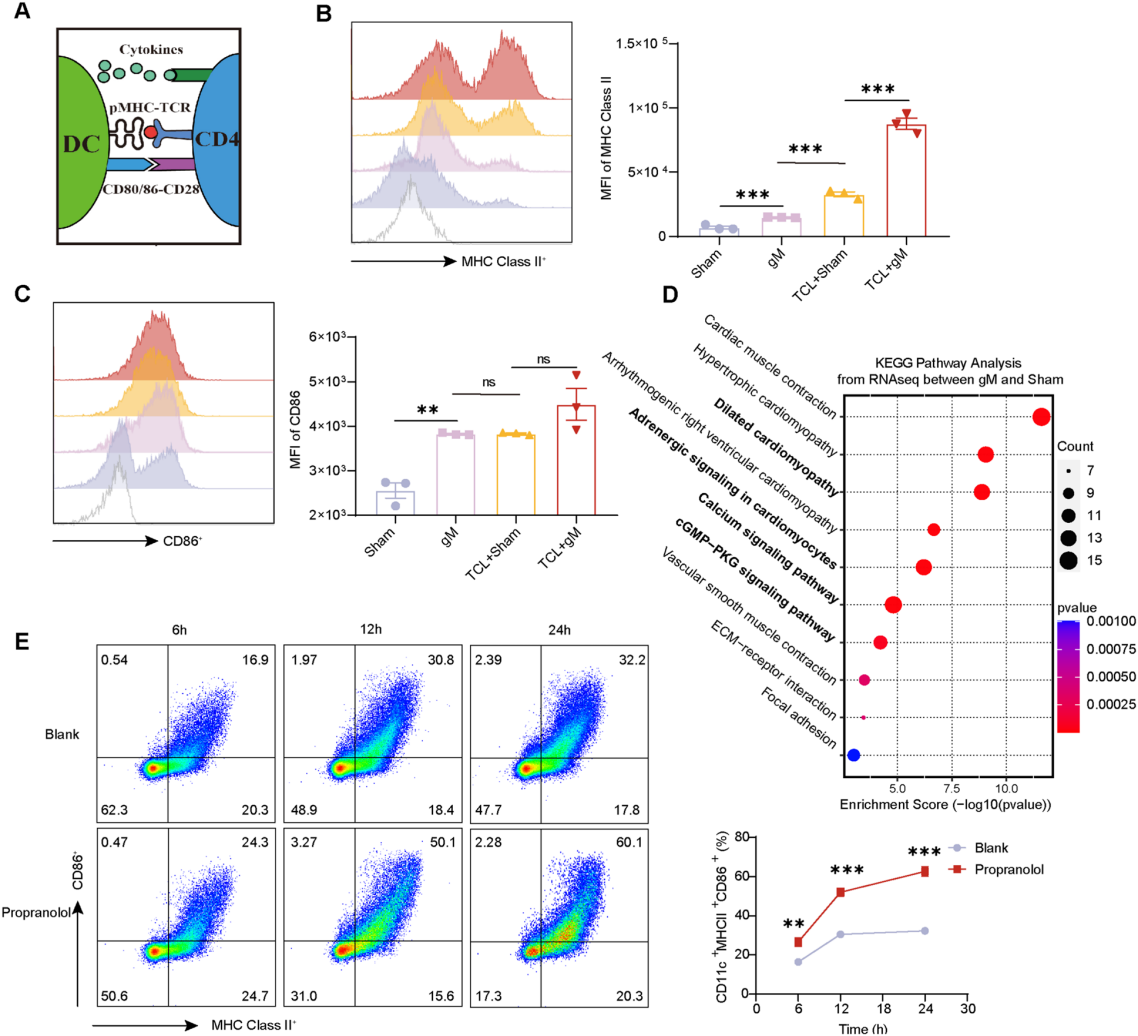
The combination of gM with the TCL vaccine promotes DC activation in paracancerous lymph nodes by inhibiting the adrenergic signalling pathway. **A** Schematic outline of the interaction between dendritic cells and CD4^+^ T cells. **B** Flow cytometry analysis of MHC class II^+^ activation markers in CD11c^+^ dendritic cells from paracancerous lymph nodes from the Sham, gM, TCL + Sham and TCL + gM groups (n = 3 mice per group); **C** Flow cytometry analysis of the CD86 activation marker in CD11c^+^ dendritic cells of paracancerous lymph nodes from the Sham, gM, TCL + Sham and TCL + gM groups (n = 3 mice per group). **D** KEGG pathway analysis of RNA-seq data [26, 43–45] from tumors in the Sham and gM groups (n = 4 mice per group). Downstream pathways of the adrenergic receptor family are marked in bold. **E** Flow cytometry analysis of the percentage of MHC class II^+^CD86^+^ CD11c^+^ bone marrow-derived dendritic cells (BMDCs) treated with/without propranolol for 6–24 h. “ns”: no statistical significance. * *p* < 0.05; ** *p* < 0.01; *** *p* < 0.001

Tumor antigens are typically not recognized by T cells directly; they require uptake and presentation by DC for effective T cell activation. Upon engagement with peptide-Major Histocompatibility Complex class II complexes (MHC-II), along with the interaction of costimulatory molecules CD80/CD86 and cytokines produced by DC, naive CD4^+^ T cells are activated and differentiate into effector T cells (Fig. 5A). Essentially, the functionality of CD4^+^ T cells is often contingent upon the activity of DC [41, 42]. To investigate whether gM facilitates the activation of CD4^+^ T cells through modulation of DC, we examined the expression of activation markers on DC within tumor-adjacent lymph nodes. Our findings revealed that gM administration alone significantly increased the expression of MHC-II and CD86 on DC. Additionally, the concurrent application of gM and the TCL vaccine resulted in even higher levels of MHC-II on DC (Fig. 5B, C), indicating that gM plays a crucial role in the activation of DC.

To elucidate the mechanism by which gM regulates DC function, we conducted RNA-sequencing on tumor samples from control and gM-treated groups, followed by KEGG signaling pathway [43–45] analysis of the differentially expressed genes. Notably, among the top 10 enriched signaling pathways, four were found to be down-regulated and associated with the β-adrenergic receptor pathway, implying that gM may suppress β-adrenergic receptor downstream signaling (Fig. 5D).

Propranolol, a well-known β-adrenergic receptor inhibitor, was utilized to test the hypothesis that gM activates DC by inhibiting β-adrenergic receptor signaling. Indeed, treatment of DC with propranolol for 6 h, 12 h, and 24 h in vitro led to an up-regulation of DC activation, as evidenced by increased expression of activation markers (Fig. 5E). And We also compared the effects of the β-agonist epinephrine on DC and found that it impairs DC maturation(Figure S3B-C). These results suggest that gM may activate DC through the modulation of β-adrenergic receptor signaling.

### Propranolol enhances the anti-tumor effect of the TCL vaccine

**Fig. 6:**
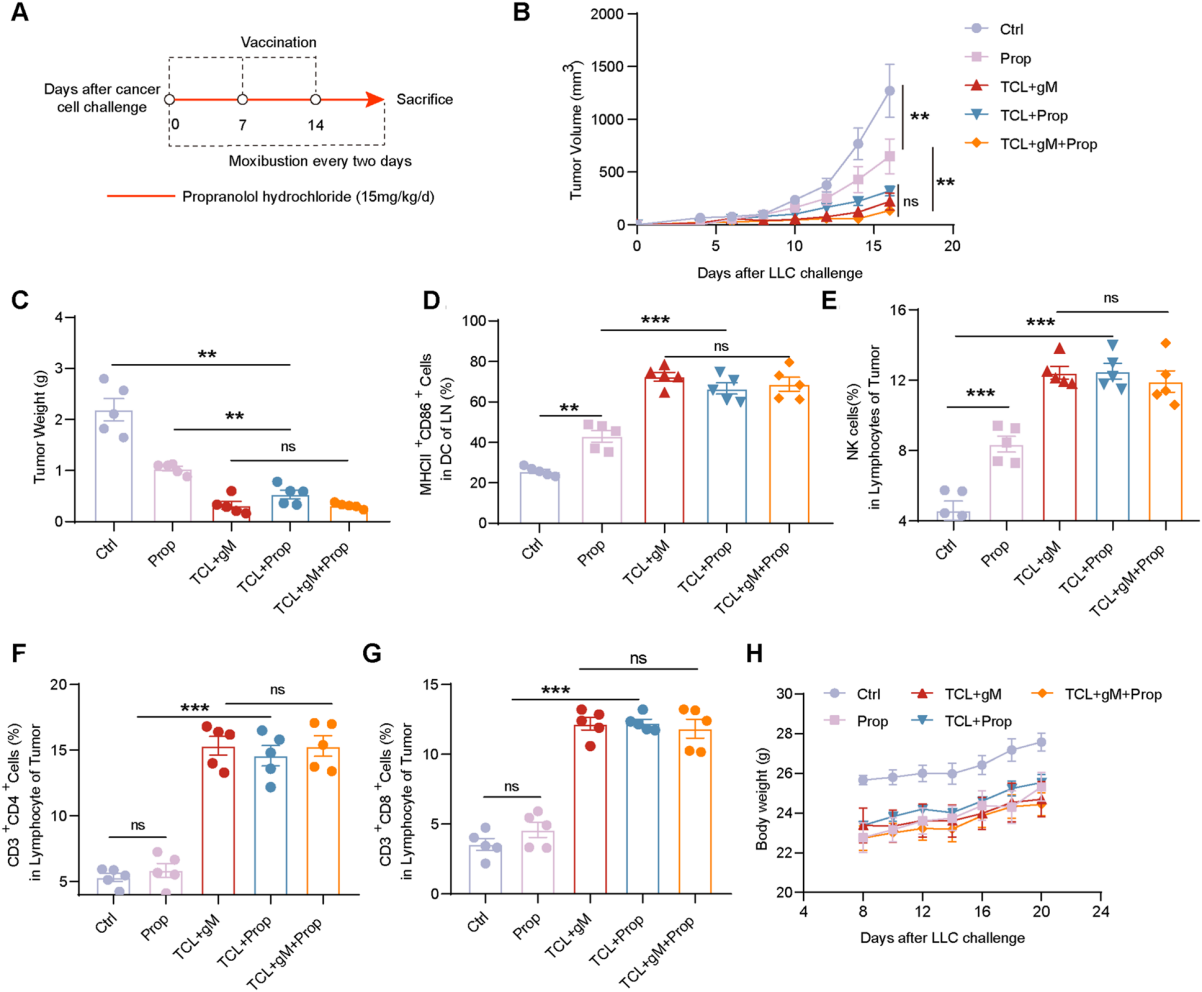
The combination of propranolol with the TCL vaccine enhances antitumor immunity. **A** Schematic outline of propranolol (Prop) used in this study; **B**, **C** Tumor growth curves/tumor weights of the Ctrl, Prop, TCL + gM, TCL + Prop and TCL + gM + Prop groups (n = 5 mice per group); **D** Flow cytometry analysis of the percentage of MHC class II^+^CD86^+^CD11c^+^ dendritic cells in the paracancerous lymph nodes of the Ctrl, Prop, TCL + gM, TCL + Prop and TCL + gM + Prop groups (n = 5 mice per group); **E–G** Flow cytometry analysis of the percentages of NK, CD4^+^T and CD8^+^ T cells in the lymphocytes of tumors from the Ctrl, Prop, TCL + gM, TCL + Prop and TCL + gM + Prop groups (n = 5 mice per group); **H** Body weights of the mice from the Ctrl, Prop, TCL + gM, TCL + Prop and TCL + gM + Prop groups (n = 5 mice per group). “ns”: no statistical significance. * *p* < 0.05; ** *p* < 0.01; *** *p* < 0.001

Given that the effects of propranolol resemble those of gM, we postulated that the combination of propranolol with a TCL vaccine might elicit a more robust anti-tumor immune response. To test this hypothesis, tumor-bearing mice were vaccinated and concurrently administered daily intraperitoneal injections of propranolol (Fig. 6A). We then monitored tumor growth in the mice and compared the outcomes between the TCL + gM group and the TCL + gM + prop group. The findings indicated that the TCL + prop group exhibited a comparable antitumor effect to that of the TCL + gM group, suggesting that propranolol can also potentiate the efficacy of the TCL vaccine (Fig. 6B, C).

Consistent with these observations, we employed flow cytometry to assess the proportions of mature DC in paracancerous lymph nodes and the infiltration of NK, CD4^+^, and CD8^+^ T cells within the tumor microenvironment across all groups. The data revealed that both propranolol and gM similarly enhanced the levels of these immune cells (Fig. 6D–G). Moreover, propranolol did not induce significant toxicity when administered alone or in combination with gM (Fig. 6H), indicating that propranolol may serve as a potential adjuvant for cancer vaccines.

## Discussion

Poor clinical outcomes represent a significant challenge for cancer vaccines, and there is an urgent need to develop novel strategies to enhance their efficacy. In this study, we discovered that gM can inhibit the β-adrenergic receptor signaling pathway and augment DC function, thereby potentiating the antitumor immune response elicited by cancer vaccines and ultimately suppressing tumor progression.

Previous research has demonstrated that stimulation of β-adrenoceptors compromises the functionality of DC and cytotoxic CD8^+^ T lymphocytes [46–48]. The dysfunction of DC and CD8^+^ T cells, which serve as primary effector cells, likely contributes to the ineffectiveness of cancer vaccines. Therefore, inhibiting β-adrenoceptors may enhance vaccine efficacy. Several studies have demonstrated that the β-adrenoceptor antagonist propranolol can potentiate the effects of tumor vaccines when used as an adjuvant. For instance, Somayeh Ashrafi et al. reported that the combination of propranolol with a tumor vaccine suppresses tumor growth by modulating cytokine patterns within the tumor microenvironment [49]; Clara Daher et al. also demonstrated that propranolol can strongly improve the efficacy of an antitumor STxBE7 vaccine by enhancing the frequency of CD8^+^ T lymphocytes infiltrating the tumor (TIL) [46]. In this study, we also demonstrated that propranolol can be a good adjuvant for the TCL vaccine.

As a nonselective β-adrenergic receptor inhibitor, propranolol exhibits significant antitumor efficacy both as a monotherapy and in combination with vaccines. However, its clinical application is constrained by adverse effects (e.g., bradycardia) and contraindications (e.g., asthma) [50, 51]. As a kind of external treatment, moxibustion does not show these adverse effects, suggesting it may be a safer, more cost-effective adjuvant for tumor vaccines with potential for rapid clinical application.

Despite the established recognition of CD8^+^ T cells as predominant effector cells in numerous vaccine studies [52–54], their contribution was comparatively limited in this study (Fig. 4D–E). This phenomenon may be attributable to variations in tumor types, vaccine designs, or individual immune responses. Although we detected the upregulation of CD8^+^ T cells in the tumor and spleen under the combination therapy, its depletion did not affect tumor growth. Moreover, NK cell depletion partly impaired the antitumor effect of the combined therapy, which implies that NK cells play an important role in vaccine therapy [55, 56]. For CD4^+^ T cells, some studies have reported a role for neoantigen-specific CD4^+^ T-cell responses in direct tumor clearance [57–59], and CD4^+^ T cells can exert direct antitumor effects independent of CD8^+^ T cells [60, 61]. These studies revealed that cytotoxic CD4^+^ T cells, which are involved in infection and autoimmunity, play a key role in tumors [62]. Our findings underscore the critical role of CD4^+^ T cells as pivotal antitumor effector cells in specific contexts, offering novel insights for further investigation into the underlying mechanisms.

In general, exogenous antigens such as TCL necessitate the presentation by mature DC to activate CD4^+^ T cells [63, 64]. In this study, we demonstrated that gM promotes DC maturation by inhibiting β-adrenoceptor signaling. However, two key questions remain unresolved. First, the precise mechanism by which gM inhibits the β-signaling pathway is not fully elucidated. While we conducted RNA sequencing of tumor tissues and used propranolol as a substitute for gM to indirectly support our hypothesis, the underlying molecular mechanisms require further investigation. Considering that accupoint therapy typically involves regulation of the nervous system, our subsequent investigation aims to explore whether moxibustion mediates the downregulation of adrenergic receptor signaling pathways by modulating neurotransmitter secretion through the sympathetic nervous system. Second, the impact of the β-signaling pathway on DC maturation remains unclear. Classical downstream components protein kinase A had little effects on DC differentiation While inhibition of NF-κB had samiliar effect as propranolol (Figure S3B-C). The specific mechanisms warrant additional exploration.

## Supplementary Information

Additional file1 (DOCX 720 KB)

## Data Availability

The authors confirm that the data supporting the findings of this study are available within the article.

## Abbreviations

APC: Antigen-presenting cells
DC: Dendritic cell
DCV: DC vaccines
gM: Grain-sized moxibustion
LLC: Lewis Lung Carcinoma
MHC-II: Major histocompatibility complex class II complexes
NK: Natural killer cells
PBS: Phosphate-buffered saline
Prop: Propranolol hydrochloride
SEM: Standard errors of the means
SPF: Specific pathogen-free
ST 36: Zusanli acupoints
TCL: Tumor cell lysate vaccines
TCM: Traditional Chinese medicine

## References

[1] Liu J, Fu M, Wang M, Wan D, Wei Y, Wei X. Cancer vaccines as promising immuno-therapeutics: platforms and current progress. J Hematol Oncol. 2022;15(1):28. 35303904 10.1186/s13045-022-01247-x PMC8931585

[2] Zhao S, Wu S, Jiang S, Zhao G, Wang B. Developing effective cancer vaccines using rendered-inactive tumor cells. Vaccines (Basel). 2023;11(8):1330. 37631898 10.3390/vaccines11081330 PMC10458160

[3] Salewski I, Gladbach YS, Kuntoff S, Irmscher N, Hahn O, Junghanss C, et al. In vivo vaccination with cell line-derived whole tumor lysates: neoantigen quality, not quantity matters. J Transl Med. 2020;18(1):402. 33087163 10.1186/s12967-020-02570-y PMC7579816

[4] Maruggi G, Zhang C, Li J, Ulmer JB, Yu D. mRNA as a transformative technology for vaccine development to control infectious diseases. Mol Ther. 2019;27(4):757–72. 30803823 10.1016/j.ymthe.2019.01.020 PMC6453507

[5] Neefjes J, Ovaa H. A peptide’s perspective on antigen presentation to the immune system. Nat Chem Biol. 2013;9(12):769–75. 24231618 10.1038/nchembio.1391

[6] Cazet A, Julien S, Bobowski M, Burchell J, Delannoy P. Tumour-associated carbohydrate antigens in breast cancer. Breast Cancer Res. 2010;12(3):204. 20550729 10.1186/bcr2577 PMC2917018

[7] Morse MA, Gwin WR 3rd, Mitchell DA. Vaccine therapies for cancer: then and now. Target Oncol. 2021;16(2):121–52. 33512679 10.1007/s11523-020-00788-w PMC7845582

[8] Yarchoan M, Gane EJ, Marron TU, Perales-Linares R, Yan J, Cooch N, et al. Personalized neoantigen vaccine and pembrolizumab in advanced hepatocellular carcinoma: a phase 1/2 trial. Nat Med. 2024;30(4):1044–53. 38584166 10.1038/s41591-024-02894-y PMC11031401

[9] Zhao T, Cai Y, Jiang Y, He X, Wei Y, Yu Y, et al. Vaccine adjuvants: mechanisms and platforms. Signal Transduct Target Ther. 2023;8(1):283. 37468460 10.1038/s41392-023-01557-7 PMC10356842

[10] Gong N, Alameh MG, El-Mayta R, Xue L, Weissman D, Mitchell MJ. Enhancing in situ cancer vaccines using delivery technologies. Nat Rev Drug Discov. 2024;23(8):607–25. 38951662 10.1038/s41573-024-00974-9

[11] Lang F, Schrörs B, Löwer M, Türeci Ö, Sahin U. Identification of neoantigens for individualized therapeutic cancer vaccines. Nat Rev Drug Discov. 2022;21(4):261–82. 35105974 10.1038/s41573-021-00387-y PMC7612664

[12] Sahin U, Oehm P, Derhovanessian E, Jabulowsky RA, Vormehr M, Gold M, et al. An RNA vaccine drives immunity in checkpoint-inhibitor-treated melanoma. Nature. 2020;585(7823):107–12. 32728218 10.1038/s41586-020-2537-9

[13] Shibata T, Lieblong BJ, Sasagawa T, Nakagawa M. The promise of combining cancer vaccine and checkpoint blockade for treating HPV-related cancer. Cancer Treat Rev. 2019;78:8–16. 31302573 10.1016/j.ctrv.2019.07.001 PMC6710123

[14] Vermaelen K. Vaccine strategies to improve anti-cancer cellular immune responses. Front Immunol. 2019;10:8. 30723469 10.3389/fimmu.2019.00008 PMC6349827

[15] Zhang WB, Gao YT, Li HY. Analysis of compiling date of Huang di neijing (Huangdi’s internal classic). Zhonghua Yi Shi Za Zhi. 2017;47(3):173–7. 28810350 10.3760/cma.j.issn.0255-7053.2017.03.009

[16] Li S, Fu H, Ju B. First discrimination of the meanings of the seven words relevant with acupuncture in Huang di Neijing (Yellow Emnerors internal classic). Zhongguo Zhen Jiu. 2015;35(10):1080–2. 26790230

[17] Hsieh C-H, Tseng S-T, Hung Y-C, Chang T-C, Hu W-L, Lin C-H. Effect of moxibustion on meridian in a warm needling model: a protocol for a prospective observational study. Medicine (Baltimore). 2022;101(47): e31492. 36451391 10.1097/MD.0000000000031492 PMC9704875

[18] Yao W-J, Liu P-Z, Fan Y-L, Zhang Z-C, Guo X-D, Gao X-Y. Adjuvant treatment of penetrating moxibustion at governor vessel for persistent allergic rhinitis of deficiency-cold syndrome. Zhongguo Zhen Jiu. 2021;41(6):623–7. 34085478 10.13703/j.0255-2930.20201229-0002

[19] Wang L-L. Characteristic of moxibustion and its warming-dredging effect. Zhongguo Zhen Jiu. 2011;31(10):865–8. 22043667

[20] Deng H, Shen X. The mechanism of moxibustion: ancient theory and modern research. Evid Based Complem Alternat Med. 2013;2013: 379291. 10.1155/2013/379291 PMC3789413 24159344

[21] Zhou Z-h, Yuan Y-q. Survey of Japan moxibustion methods. Zhongguo Zhen Jiu. 2008;28(1):65–7. 18257194

[22] Huang Q-F, Xie C, Wu H-G, Yang G, Liu J, Guo X-C, et al. Spectrum and indications of acupuncture and moxibustion therapy based on bibliometric analysis. Zhongguo Zhen Jiu. 2021;41(9):1055–9. 34491658 10.13703/j.0255-2930.20200818-0002

[23] Choi TY, Kim TH, Kang JW, Lee MS, Ernst E. Moxibustion for rheumatic conditions: a systematic review and meta-analysis. Clin Rheumatol. 2011;30(7):937–45. 21331532 10.1007/s10067-011-1706-5

[24] Lee DH, Kim JI, Lee MS, Choi TY, Choi SM, Ernst E. Moxibustion for ulcerative colitis: a systematic review and meta-analysis. BMC Gastroenterol. 2010;10:36. 20374658 10.1186/1471-230X-10-36 PMC2864201

[25] Qi Q, Liu Y-N, Jin X-M, Zhang L-S, Wang C, Bao C-H, et al. Moxibustion treatment modulates the gut microbiota and immune function in a dextran sulphate sodium-induced colitis rat model. World J Gastroenterol. 2018;24(28):3130–44. 30065559 10.3748/wjg.v24.i28.3130 PMC6064969

[26] Hu D, Shen W, Gong C, Fang C, Yao C, Zhu X, et al. Grain-sized moxibustion promotes NK cell antitumour immunity by inhibiting adrenergic signalling in non-small cell lung cancer. J Cell Mol Med. 2021;25(6):2900–8. 33506637 10.1111/jcmm.16320 PMC7957214

[27] Lu S, Wang B, Wang J, Guo Y, Li S, Zhao S, et al. Moxibustion for the treatment of cancer and its complications: efficacies and mechanisms. Integr Cancer Ther. 2023;22:15347354231198088. 37746720 10.1177/15347354231198089 PMC10521285

[28] Galon J, Bruni D. Approaches to treat immune hot, altered and cold tumours with combination immunotherapies. Nat Rev Drug Discov. 2019;18(3):197–218. 30610226 10.1038/s41573-018-0007-y

[29] Wu B, Zhang B, Li B, Wu H, Jiang M. Cold and hot tumors: from molecular mechanisms to targeted therapy. Signal Transduct Target Ther. 2024;9(1):274. 39420203 10.1038/s41392-024-01979-x PMC11491057

[30] Xu B, Zhang X, Li J, Han X, Zhu X. Interpretation of the cause, mechanism, syndrome, and treatment of “cold” in the five stages of tumor evolution: “Deficiency-Cold-Toxin-Obstruction-Decline.” Glob Tradit Chin Med. 2023;16(7):1407–11.

[31] Jie L. Five-stage evolution: construction and innovation of the theoretical system of traditional Chinese medicine in prevention and treatment of malignant tumors. J Beijing Univ Tradit Chin Med. 2022;45(3):223–30.

[32] Ji Y, Li S, Zhang X, Liu Y, Lu Q, Li Q, et al. Impact of chemotherapy on traditional chinese medicine constitution in breast cancer patients and the regulatory role of moxibustion. World J Integr Tradit Western Med. 2020;15(04):735–41.

[33] Bertram JS, Janik P. Establishment of a cloned line of Lewis Lung Carcinoma cells adapted to cell culture. Cancer Lett. 1980;11(1):63–73. 7226139 10.1016/0304-3835(80)90130-5

[34] Inaba K, Inaba M, Romani N, Aya H, Deguchi M, Ikehara S, et al. Generation of large numbers of dendritic cells from mouse bone marrow cultures supplemented with granulocyte/macrophage colony-stimulating factor. J Exp Med. 1992;176(6):1693–702. 1460426 10.1084/jem.176.6.1693 PMC2119469

[35] Lin TJ, Lin HT, Chang WT, Mitapalli SP, Hsiao PW, Yin SY, et al. Shikonin-enhanced cell immunogenicity of tumor vaccine is mediated by the differential effects of DAMP components. Mol Cancer. 2015;14:174. 26403780 10.1186/s12943-015-0435-9 PMC4582891

[36] Han J, Kim S, Hwang YH, Kim SA, Lee Y, Kim J, et al. Novel personalized cancer vaccine using tumor extracellular vesicles with attenuated tumorigenicity and enhanced immunogenicity. Adv Sci (Weinh). 2024;11(25): e2308662. 38666427 10.1002/advs.202308662 PMC11220679

[37] Felix NJ, Donermeyer DL, Horvath S, Walters JJ, Gross ML, Suri A, et al. Alloreactive T cells respond specifically to multiple distinct peptide-MHC complexes. Nat Immunol. 2007;8(4):388–97. 17322886 10.1038/ni1446

[38] Shih FF, Racz J, Allen PM. Differential MHC class II presentation of a pathogenic autoantigen during health and disease. J Immunol. 2006;176(6):3438–48. 16517712 10.4049/jimmunol.176.6.3438

[39] Bomber P, McCready R, Hammersley P. Propranolol hydrochloride enhancement of tumor perfusion and uptake of gallium-67 in a mouse sarcoma. J Nuclear Med. 1986;27(2):243–5. 3712042

[40] Speiser DE, Chijioke O, Schaeuble K, Munz C. CD4(+) T cells in cancer. Nat Cancer. 2023;4(3):317–29. 36894637 10.1038/s43018-023-00521-2

[41] Liu J, Zhang X, Cheng Y, Cao X. Dendritic cell migration in inflammation and immunity. Cell Mol Immunol. 2021;18(11):2461–71. 34302064 10.1038/s41423-021-00726-4 PMC8298985

[42] Hilligan KL, Ronchese F. Antigen presentation by dendritic cells and their instruction of CD4+ T helper cell responses. Cell Mol Immunol. 2020;17(6):587–99. 32433540 10.1038/s41423-020-0465-0 PMC7264306

[43] Kanehisa M, Furumichi M, Sato Y, Matsuura Y, Ishiguro-Watanabe M. KEGG: biological systems database as a model of the real world. Nucl Acids Res. 2025;53(D1):D672–7. 39417505 10.1093/nar/gkae909 PMC11701520

[44] Kanehisa M. Toward understanding the origin and evolution of cellular organisms. Protein Sci. 2019;28(11):1947–51. 31441146 10.1002/pro.3715 PMC6798127

[45] Kanehisa M, Goto S. KEGG: kyoto encyclopedia of genes and genomes. Nucleic Acids Res. 2000;28(1):27–30. 10592173 10.1093/nar/28.1.27 PMC102409

[46] Daher C, Vimeux L, Stoeva R, Peranzoni E, Bismuth G, Wieduwild E, et al. Blockade of beta-adrenergic receptors improves CD8(+) T-cell priming and cancer vaccine efficacy. Cancer Immunol Res. 2019;7(11):1849–63. 31527069 10.1158/2326-6066.CIR-18-0833

[47] Herve J, Dubreil L, Tardif V, Terme M, Pogu S, Anegon I, et al. beta2-Adrenoreceptor agonist inhibits antigen cross-presentation by dendritic cells. J Immunol. 2013;190(7):3163–71. 23420884 10.4049/jimmunol.1201391

[48] Herve J, Haurogne K, Bacou E, Pogu S, Allard M, Mignot G, et al. beta2-adrenergic stimulation of dendritic cells favors IL-10 secretion by CD4(+) T cells. Immunol Res. 2017;65(6):1156–63. 29134568 10.1007/s12026-017-8966-3

[49] Ashrafi S, Shapouri R, Mahdavi M. Immunological consequences of immunization with tumor lysate vaccine and propranolol as an adjuvant: a study on cytokine profiles in breast tumor microenvironment. Immunol Lett. 2017;181:63–70. 27899275 10.1016/j.imlet.2016.11.014

[50] Poyurovsky M, Weizman A. Treatment of antipsychotic-induced akathisia: role of serotonin 5-HT(2a) receptor antagonists. Drugs. 2020;80(9):871–82. 32385739 10.1007/s40265-020-01312-0

[51] Ji Y, Chen S, Wang Q, Xiang B, Xu Z, Zhong L, et al. Intolerable side effects during propranolol therapy for infantile hemangioma: frequency, risk factors and management. Sci Rep. 2018;8(1):4264. 29523832 10.1038/s41598-018-22787-8 PMC5844887

[52] Baharom F, Ramirez-Valdez RA, Khalilnezhad A, Khalilnezhad S, Dillon M, Hermans D, et al. Systemic vaccination induces CD8(+) T cells and remodels the tumor microenvironment. Cell. 2022;185(23):4317–32. 36302380 10.1016/j.cell.2022.10.006 PMC9669246

[53] Baharom F, Ramirez-Valdez RA, Tobin KKS, Yamane H, Dutertre CA, Khalilnezhad A, et al. Intravenous nanoparticle vaccination generates stem-like TCF1(+) neoantigen-specific CD8(+) T cells. Nat Immunol. 2021;22(1):41–52. 33139915 10.1038/s41590-020-00810-3 PMC7746638

[54] Park KS, Nam J, Son S, Moon JJ. Personalized combination nano-immunotherapy for robust induction and tumor infiltration of CD8(+) T cells. Biomaterials. 2021;274: 120844. 33962217 10.1016/j.biomaterials.2021.120844 PMC8184601

[55] Diniz MO, Schurich A, Chinnakannan SK, Duriez M, Stegmann KA, Davies J, et al. NK cells limit therapeutic vaccine-induced CD8(+)T cell immunity in a PD-L1-dependent manner. Sci Transl Med. 2022;14(640):eabi4670. 35417187 10.1126/scitranslmed.abi4670 PMC7619356

[56] Badrinath S, Dellacherie MO, Li A, Zheng S, Zhang X, Sobral M, et al. A vaccine targeting resistant tumours by dual T cell plus NK cell attack. Nature. 2022;606(7916):992–8. 35614223 10.1038/s41586-022-04772-4 PMC10253041

[57] Tran E, Turcotte S, Gros A, Robbins PF, Lu YC, Dudley ME, et al. Cancer immunotherapy based on mutation-specific CD4+ T cells in a patient with epithelial cancer. Science. 2014;344(6184):641–5. 24812403 10.1126/science.1251102 PMC6686185

[58] Schumacher T, Bunse L, Pusch S, Sahm F, Wiestler B, Quandt J, et al. A vaccine targeting mutant IDH1 induces antitumour immunity. Nature. 2014;512(7514):324–7. 25043048 10.1038/nature13387

[59] Kreiter S, Vormehr M, van de Roemer N, Diken M, Lower M, Diekmann J, et al. Mutant MHC class II epitopes drive therapeutic immune responses to cancer. Nature. 2015;520(7549):692–6. 25901682 10.1038/nature14426 PMC4838069

[60] Spitzer MH, Carmi Y, Reticker-Flynn NE, Kwek SS, Madhireddy D, Martins MM, et al. Systemic immunity is required for effective cancer immunotherapy. Cell. 2017;168(3):487–502. 28111070 10.1016/j.cell.2016.12.022 PMC5312823

[61] Hirschhorn-Cymerman D, Budhu S, Kitano S, Liu C, Zhao F, Zhong H, et al. Induction of tumoricidal function in CD4+ T cells is associated with concomitant memory and terminally differentiated phenotype. J Exp Med. 2012;209(11):2113–26. 23008334 10.1084/jem.20120532 PMC3478933

[62] Oh DY, Fong L. Cytotoxic CD4(+) T cells in cancer: expanding the immune effector toolbox. Immunity. 2021;54(12):2701–11. 34910940 10.1016/j.immuni.2021.11.015 PMC8809482

[63] Bottcher JP, Reis e Sousa C. The role of type 1 conventional dendritic cells in cancer immunity. Trends Cancer. 2018;4(11):784–92. 30352680 10.1016/j.trecan.2018.09.001 PMC6207145

[64] Wculek SK, Cueto FJ, Mujal AM, Melero I, Krummel MF, Sancho D. Dendritic cells in cancer immunology and immunotherapy. Nat Rev Immunol. 2020;20(1):7–24. 31467405 10.1038/s41577-019-0210-z

