# Supplementary material for: Grain-sized moxibustion activates dendritic cells to enhance the antitumor immunity of cancer vaccines
Source: Chin Med. 2025 May 27;20:73. doi: 10.1186/s13020-025-01134-w (PMC12107723; doi:10.1186/s13020-025-01134-w)
Supplement: Supplementary file 1 — Additional file1 (DOCX 720 KB) [file 13020_2025_1134_MOESM1_ESM.docx]

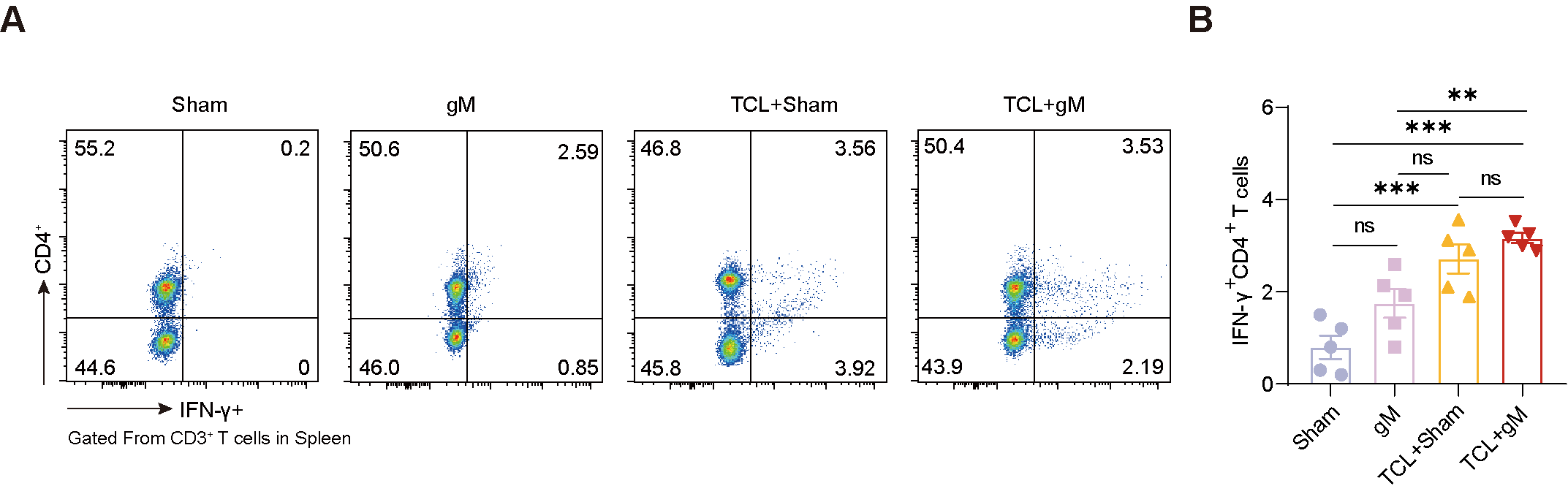


**Figure S1. The combination of gM with cancer vaccines enhances the immune response**

**A-B.** Flow cytometry analysis of the percentage of IFN-γ^+^CD4^+^ T cells in the spleens of mice from the group Sham, gM, TCL+Sham and TCL+gM (n=5 per group). “ns”: not statistical significance. * p < 0.05; ** p < 0.01; *** p < 0.001.


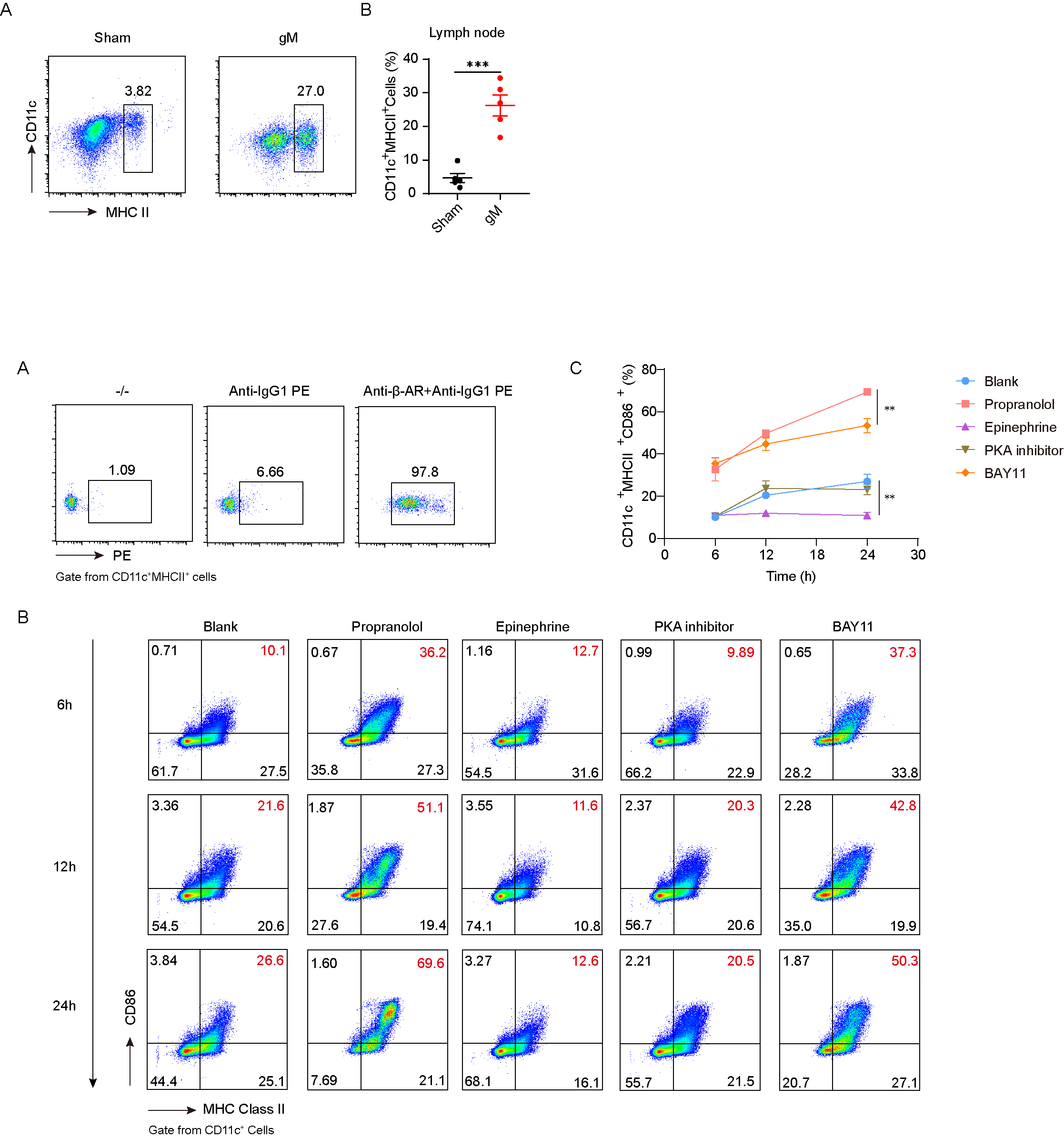


**Figure S2. gM increases the percent of mature dendritic cells in the paracancerous lymph nodes.**

**A-B.** Flow cytometry analysis of the percentage of CD11c^+^MHC II^+^ cells in the paracancerous lymph nodes of mice from the group Sham and gM (n=5 per group). *** p < 0.001.


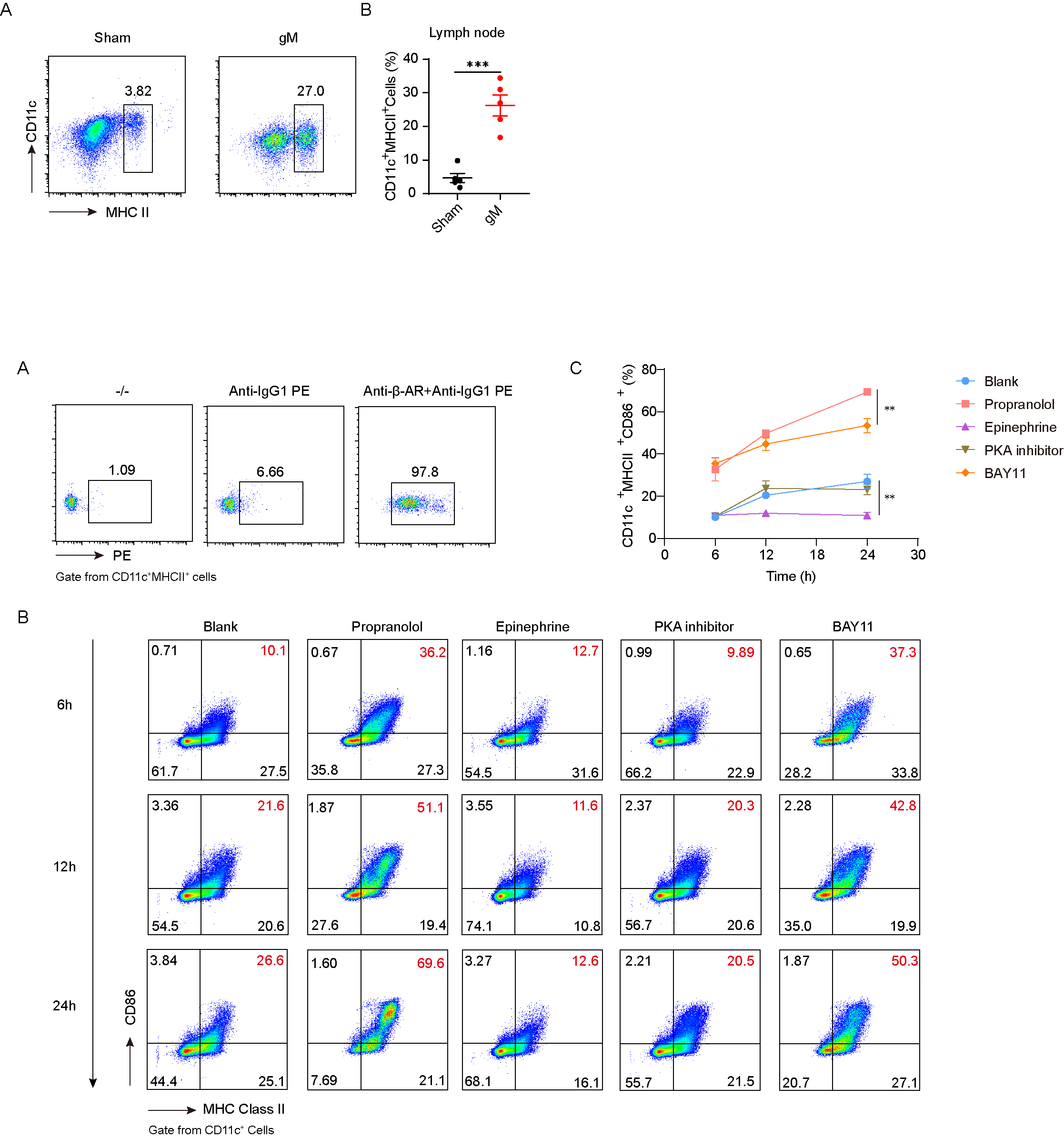


**Figure S3. Inhibiton of β-AR promotes the maturation of dendritic cells.**

**A.** The expression of β-AR on the surface of CD11c^+^MHC II^+^ cells; **B-C.** the effect of different treatment to the maturation of DC. Blank:Untreated; Propranolol: inhibition of β-AR; Epinephrine: Activation of β-AR; PKA : a classic downstream protein of β-AR pathway; BAY11：a inhibitor of NF-κB pathway. ** p < 0.01.
